# A Protocol for the Prospective Evaluation of Novel Suction-Based Airway Clearance Devices in the Treatment of Foreign Body Airway Obstructions

**DOI:** 10.7759/cureus.20918

**Published:** 2022-01-04

**Authors:** Cody L Dunne, Catarina Queiroga, David Szpiman, Kayla Viguers, Selana Osman, Amy E Peden

**Affiliations:** 1 Department of Emergency Medicine, University of Calgary, Calgary, CAN; 2 Epidemiology Research Unit, Instituto de Saúde Pública da Universidade do Porto, Porto, PRT; 3 Sociedade Brasileira de Salvamento Aquático, Brazilian Lifesaving Society, Rio de Janeiro, BRA; 4 Faculty of Medicine, Memorial University of Newfoundland, St. John's, CAN; 5 Cumming School of Medicine, University of Calgary, Calgary, CAN; 6 School of Population Health, Faculty of Medicine, University of New South Wales, Sydney, AUS

**Keywords:** qualitative research, theoretical domains framework, prospective study, dechoker, lifevac, anti-choking device, foreign body airway obstruction, choking

## Abstract

Background

Foreign body airway obstructions (FBAOs, choking) are a significant cause of preventable mortality. Abdominal thrusts, back blows, and chest compressions are traditional interventions. However, suction-based airway clearance devices (ACDs) have recently been marketed as an alternative. Of note, there is limited published evidence regarding their efficacy and safety. Our research has two aims: (1) to investigate what situational and patient factors are frequently identified, and which are associated with relief of the FBAO and survival in individuals with FBAOs treated with an ACD; and (2) to describe the experience of individuals who have used ACDs in response to a FBAO and identify facilitators and barriers to the use of ACDs compared to traditional interventions.

Methods and analysis

A prospective database will be developed using an online reporting system to capture ACD uses, independent of manufacturers, from July 1st, 2021 to December 31st, 2023. Descriptive statistics will be used to summarize cases, outcomes, and adverse events. Clinically important subgroups will be stratified for analysis, including the severity of obstruction, patient demographics, and training of ACD users. Semi-structured interviews will also be conducted with a subset of ACD users to describe in detail their experience using the device. Themes from these interviews will be assessed using the theoretical domains framework.

Discussion

This study will improve the evidence surrounding ACDs and compare it to current data for traditional techniques, with the aim of optimizing FBAO treatment. Data on ACDs are urgently needed as these devices are already being used by parents, caregivers, lay rescuers, and healthcare professionals to respond to choking emergencies. This evaluation will provide important information about their effectiveness and any safety concerns which can inform the public, resuscitation guidelines, and future research studies.

## Introduction

Foreign body airway obstructions (FBAOs, choking) are preventable injuries with a considerable mortality burden globally [1-5]. The outcome of a FBAO is determined largely by bystander actions taken outside of the hospital setting, and yet there is a paucity of contemporary data informing the best response to be undertaken by lay rescuers and first responders [6].

The recent systematic review, authored by Couper et al., best summarizes the guiding literature in this area and highlights the limitations of current data [6]. Current recommendations from resuscitation councils, influenced by the International Liaison Committee on Resuscitation’s (ILCOR) Consensus on Science with Treatment Recommendations (CoSTR), suggest a combination of abdominal thrusts, back blows, and chest compressions as the core interventions for FBAO [7]. Notably, the majority of data are almost entirely case series, retrospective in nature, and no studies compare which intervention is superior in specific settings.

The limitations of this data reflect the challenges of conducting rigorous research in the field of resuscitation, especially in the pre-hospital environment. FBAOs are relatively rare events, and it is nearly impossible to conduct randomized studies where interventions are assigned as the vast majority are treated before emergency medical service (EMS) workers arrive [8]. Further, reliance on only healthcare records (either from EMS or physicians) may omit nuances in situational factors or interventions that researchers are trying to evaluate. Conversely, data acquired from the memory of the patient or bystanders are subject to recall bias and are likely influenced by poor medical literacy [9]. This makes the data collected more limited and of lower quality compared to other areas of medical research. It also blurs the standard that new interventions must meet before being deemed safe and, subsequently, effective.

One new FBAO intervention that has been described in the past decade is airway clearance devices (ACDs). These externally applied, non-powered, portable suction devices have been marketed as an alternative when traditional FBAO techniques fail and fall into two broad types, which, despite their similarities, have considerable differences in their design. Non-invasive ACDs are primarily manufactured by LifeVac© (LifeVac LLC, Nesconset, New York, USA), and consist of a facemask attached to a compressible bellows system [10]. Applying the mask to the choking person’s face creates a seal, followed by a rapid depression of the plunger system and a firm pull upwards. Minimally invasive ACDs are primarily manufactured by Dechoker© (Dechoker LLC, Concord, NC, USA), and consist of a short oropharyngeal tube that acts as a tongue depressor when the external face mask is applied [11]. The attached cylinder has a plunger that is pulled sharply to create the negative pressure system once a seal is formed with the mouth. Both contrast with other suction devices (e.g., Laerdal© V-Vac®) that do not have the external anchor of the face mask and can enter deeper into the oral airway [12]. Both devices offer training primarily through online videos from their websites, which can be viewed by purchasers and the public. 

However, two independent systematic reviews recently conducted concluded that there is insufficient evidence to support or discourage ACD use in the treatment of FBAO [6,13]. Both reviews only identified ten unique cases describing the safety of LifeVac’s ACD, all of which were successful at FBAO relief with no adverse events documented. They concluded with a recommendation that further evaluation in a research setting would be beneficial to determine the safety and effectiveness profile of ACDs.

Updated evidence in this area is required urgently. As sales of ACDs are increasing worldwide, the public and resuscitation councils require them to make informed decisions on their utility [10,11]. If the safety and effectiveness profile remain promising, then acknowledging ACDs as an alternative to traditional techniques is appropriate. If not, then this also needs to be clarified. Regardless, the first step in this process is to conduct research, independent of industry, examining these outcomes further.

This protocol details the present study’s proposed method to address the following objectives:

Research Aim One: To investigate what situational and patient factors are frequently identified, and which are associated with relief of the FBAO and survival in individuals with a FBAO treated with an ACD.

Research Aim Two: To describe the experiences of individuals who have used ACDs in response to a FBAO and identify facilitators and barriers to the use of ACDs compared to traditional interventions.

## Materials and methods

This is a multi-method, international observational study that will run from July 1st, 2021 to December 31st, 2023. Two research study designs will be used to address the research aims.

Study development

Following the publication of the systematic review on the effectiveness of ACDs in January 2020, the research team met in late 2020 to discuss a strategy for future investigation [13]. A draft protocol was created and underwent several revision cycles until a final version was completed and agreed upon. In early 2021, contact was made with each manufacturer to arrange a meeting where an overview of the proposed study was introduced and discussed. The CEO of each respective company, as well as several members of their administration and/or medical team, were in attendance, along with the research team. Following this, the project protocol was provided to each company to review before confirming their interest in participating. In March 2021, letters of support were signed by the CEOs of both companies, agreeing to participate to the extent that was outlined in the provided protocol, including assistance with the identification and recruitment of participants. The arms-length independence structure of the project, where manufacturers would not have any role in data collection, analysis, and reporting was reaffirmed. The study was then reviewed and approved by the Health Research Ethics Committee of the University of New South Wales (HC210242) on May 25, 2021.

Following ethics approval, a subsequent meeting was coordinated with each company with a similar cohort of representatives as the initial meeting. Here, the protocol was reaffirmed by the research team, and planning for the launch of the prospective component was discussed. A member of the research team was available to answer any questions the manufacturers had regarding the study during this period via email or phone.

Of note, no significant changes to the study protocol were made after the initial meeting with the manufacturers. A pre-print version of the a priori protocol was released on medRxiv while awaiting peer review [14].

Study one: prospective database development and evaluation

Participants and Recruitment Procedure

Participants who meet the following criteria will be eligible for inclusion in the study:

1. Individuals 18 years of age or older, who have used an ACD on a human to attempt to dislodge a FBAO. 

2. Individuals who are not cognitively impaired, with no intellectual disability or mental illness.

The only exclusion criteria outside of those not meeting the above will be anyone unwilling to provide consent or those who are non-English or Spanish-speaking (as the survey and interview guide will only be available in these languages).

ACD users will be invited to participate once they have been identified. Identification will occur in one of three methods. Both companies have agreed to include information on their webpage where users can learn about the research study and access a link to the data collection survey on an independent site if they wish to participate. Companies also agreed to notify potential participants who report ACD use to them directly via email, phone, or social media about the research study. A standardized e-mail was given to the companies to be sent to participants on behalf of the research team in this case. Finally, a website independent of all ACD manufacturers will be setup by the research team where interested individuals can learn more about the study and sign up to participate without the involvement of the manufacturers. It is believed that this will empower users who have had an unfavorable experience with ACDs to feel comfortable reporting without the stress of doing so directly to the manufacturers.

The initial electronic message sent to all interested parties will contain a link to the online survey where participants will have access to the participant information statement and consent form (PISCF) as well as the survey if they wish to proceed. At the end of the survey, participants will be able to indicate if they are interested in participating in an optional interview to help achieve Research Aim Two (see section below). 

Data Collection

Online surveys will be administered using digital survey software (Qualtrics, Provo, UT). The data collection tool was developed by the research team, and then beta-tested among ten individuals with no experience in healthcare or research. They were asked to provide feedback on the overall format of the tool as well as on any specific questions that were difficult to comprehend or were too complex to be answered in a survey format. Based on their comments, the survey was revised prior to being uploaded to the Qualtrics system.

Data will be collected in four domains: participant (ACD user), patient (choking person), situational, and outcome (Table 1). Furthermore, participants will be asked to make several agreement statements to further assess their experience.

**Table 1:** Summary of collected variables via online survey. ACD: airway clearance device; CPR: cardiopulmonary resuscitation; EMS: emergency medical services; FBAO: foreign body airway obstruction.

Data category	Variables
Participant (ACD user)	Demographics, relationship to the person who choked, previous first aid or medical training, and previous experience with airway clearance devices (e.g., training, practice, or personal use)
Patient (choking person)	Demographics, medical comorbidities, functional status pre-FBAO, and FBAO history
Situational	Date and location of FBAO, foreign body details, witnessed status, severity of FBAO, patient status at time of intervention (e.g., consciousness, crying/speaking), FBAO intervention details, ACD intervention details, CPR intervention details, and EMS activation
Outcome	Relief of FBAO, survival, survival with good neurological function, functional status post-FBAO, and adverse events

Participants will be provided with an email contact for the research team in case they are unsure how to respond to a particular question and want clarification before submitting their response. Further, all participants will be asked to consent to receiving a follow-up email in the event the research team requires clarification of their responses to ensure accuracy of responses as well as confirmation that the event occurred. Once their responses are checked by one of the research team members for clarity and follow-up is conducted if needed, the results of the survey questions will be de-identified and assigned a unique study ID.

Study two: qualitative evaluation of ACD users’ experiences

Participants and Recruitment Procedure

Participants in this study will be a subset of the individuals who participated in study one (prospective evaluation). All participants after January 1st, 2022, will be invited to consent to an optional interview further discussing their experiences with ACDs. A six-month delay will occur to ensure the research design for the prospective study is functioning effectively before integrating the optional interviews.

Data Collection

Semi-structured interviews will be conducted using either online teleconferencing software or the telephone. This will also allow data collection to be ongoing while respecting regional COVID-19 social distancing restrictions. An interview guide will be employed to facilitate the session and ensure consistency between interviewers. Interviews will be conducted by research team members trained on the interview guide. Each interviewer will have a rehearsal session with a simulated participant monitored by the primary investigator. Subsequently, they will all have their initial and randomly selected interviews monitored by another investigator to ensure a safe and high-quality experience for all involved. The interviews will be audio-recorded and then subsequently transcribed by the researchers using transcription software.

## Results

Study one: analysis of the prospective database 

Descriptive statistics will be used to summarize the demographic, situational, outcome, and adverse event data. This will include proportions, means (standard deviations), and medians (interquartile ranges) with associated 95% confidence intervals as appropriate for the variable type. 

Relief of FBAO will be defined as the resolution of choking symptoms with no further intervention (by bystanders or EMS) required. It will be a binary variable coded as "success" or "failure." Survival will be coded as alive or dead. Survival with a favorable neurological outcome will be assessed using the cerebral performance category (CPC), with a favorable outcome being a category of 1 or 2 (good or moderate) [15]. A CPC will be assigned by the researchers after reviewing participant answers about post-FBAO functional abilities. Adverse events (both patient-related and device-related) will be summarized using descriptive statistics.

One of the medical researchers (SO, KV, or CD) will assess the information for each case to ensure appropriate assignment of outcomes. All of these will be second checked by one of the physician investigators (DS or CD) to ensure accuracy and reliability. The bivariable analysis will be performed using either the student’s T (continuous) or Chi-squared test (categorical, Fisher’s exact test if n <5) to measure the association of collected data with these outcomes. In all cases, statistical significance will be defined at the level of p <0.05 for comparative analysis. 

The stratified analysis will look at clinically important subgroups including adult versus pediatric patients, mild versus severe FBAO, patients with neurological and neurocognitive impairment, type of ACD used (e.g., non-invasive versus minimally invasive), and users’ prior training (e.g., first responder/healthcare worker versus first aid trained versus layperson without training). Finally, outcomes will also be stratified by whether individuals received a single FBAO intervention (e.g., only ACDs) or multiple (e.g., back blows followed by ACDs). This is important to note because any resolution of symptoms after multiple interventions is likely due to a synergistic effect as opposed to one intervention being solely responsible.

If data are missing (or the input by the survey respondent is not usable), the proportion of missing data for each variable will be reported, and for any analysis or calculations, the sample size (n) will be clearly identified.

Study two: analysis of the ACD user interviews

The theoretical domains framework (TDF) will be used to guide the exploration of the ACD users' experience and help identify facilitators and barriers to their use [16]. If proven to be successful, the use of ACDs by first aiders will be a novel process. Most basic life support interventions (with the exception of automated external defibrillators) do not require the use of a device to intervene in a lifesaving situation. Therefore, an understanding of what behaviors facilitate or detract from the use of ACDs will complement any implementation strategies in the future by resuscitation councils and organizations.

TDF is a qualitative framework based on psychological behavior change theory and has been validated for use in the health research setting [16]. Many examples of its previous use exist in the literature. Curran et al.* *used TDF to identify barriers to the use of the Canadian CT head rules by emergency medicine practitioners [17]. Another study, by Francis et al.,* *used the same framework to understand the physicians' decision process when ordering blood transfusions [18]. Its applicability to a variety of evaluations is a strength of the framework.

After an initial 10 interviews are conducted, the data to that point will be inductively analyzed. TDF helps researchers understand behaviors by mapping themes that arise from interviews onto 14 key domains. These domains are knowledge, skills, social/professional role and identity; beliefs about capabilities; optimism; beliefs about consequences; reinforcement; intentions; goals; memory, attention and decision processes; environmental context and resources; social influences; emotion; and behavioral regulation. Two independent researchers (CD, SO, and KV) will code themes from interviews into domains of TDF and then compare them for agreement. The coders will be blinded to the type of ACD used by the participant.

Prior work utilizing the theoretical domains framework (TDF) describes a stepwise approach, and interviews will be conducted until thematic saturation has been reached [16,19]. Thematic saturation will be determined by an explicit stopping criteria.

After the initial 10 interviews are conducted, three more interviews will be conducted, analyzed, and the thematic results compared to the original 10. If new themes emerge in the last three, another interview will be conducted. The stopping criteria will be defined as when three consecutive interviews produce no new themes compared to the prior (i.e., interviews 1-11 will be compared to 12-14; then 1-12 to 13-15, and so on until no further novel themes are identified) [20].

To be clear, no new themes will have occurred when no new shared beliefs emerge (i.e., themes that two or more participants describe). Although idiosyncratic beliefs (i.e., themes only described by one participant) will be commented on in the discussion, they will not be used as a stopping criterion for thematic saturation. In the case that there is a disagreement between coders regarding specific themes, there will be a discussion between the two to try to reach consensus first. If consensus cannot be reached, a third coder will review the discrepancy and make a final decision. Agreement between coders will be measured using Cohen’s Kappa.

Data management and privacy

All survey results and interview transcripts will be encrypted and stored on a secure server with two-factor authentication maintained by the University of New South Wales. 

Prospective survey and interview data will be assigned a unique study ID, and the results will be separated from any identifying information specific to the participant. The code linking the unique study ID to the participant identification will be kept on a separate device and stored separately from the data. The code will only be accessible to the chief investigator (AP) and delegate (CD) as needed.

Audio recordings will only contain the participants’ first names, but during the transcription process, they will be replaced with the participant’s assigned unique study ID to de-identify the record. Once transcribed and validated, the audio recordings will be destroyed.

No individual patient or healthcare provider information will be released at any point. The quantitative results will be presented in aggregate form to ensure the confidentiality of the participants. Direct quotations from interviews included in publications will not involve any identifying or case-specific information.

## Discussion

These two studies will help to improve the quality of evidence on novel ACDs and will help to shape future guidelines. The results will impact the global response to FBAO and have the potential to save lives by improving the clearance rate of FBAO and stopping the progress of a choking person to cardiac arrest. By employing a multi-method design, the study will address the question of the safety and effectiveness of ACDs as well as explore the users' experience to see if the implementation of ACDs is acceptable and feasible.

However, we recognize that the planned study is not without limitations. Most significantly, is the limitation on data collection for this field. The recent systematic review, by Couperet al., presents the available literature on all FBAO interventions, and the results are summarized in Table 2 [6,7].

**Table 2:** Summary of evidence supporting current FBAO interventions from ILCOR’s most recent CoSTR. FBAO: foreign body airway obstruction; No.: number; ILCOR: International Liaison Committee on Resuscitation; CoSTR: Consensus on Science with Treatment Recommendations.

Intervention	Outcome	No. of studies and design(s)	No. of patients included
Abdominal thrusts	Relief of FBAO	Six case series	417
	Survival	Two case series	189
Back blows	Relief of FBAO	Three case series	75
	Survival	One case series	13
Chest thrusts/compressions	Relief of FBAO	One case series	28
	Survival	One observational study	138 (Only 35 received the intervention)
Airway clearance devices	Relief of FBAO	One case series	10
	Survival	One case series	10

Often surprising to those unfamiliar with resuscitation literature is the paucity of available evidence on which guidelines are based. Since FBAOs are rare events with the majority cleared before EMS arrival, rigorous research is challenging [6-8]. Due to their novelty and limited availability, even more rare are FBAOs treated with ACDs. This makes the investigation of health records from a health system looking for a cohort of patients treated with ACDs unfeasible at present. As a result, this study plans on using a self-reporting strategy.

As a sampling method, self-reporting has well-known limitations. It skews results toward extremes of outcomes (e.g., success or death) [9]. It also relies on non-medically trained personnel to report situational and outcome details, which may be prone to recall bias [9]. To address the first limitation, we acknowledge that this study will not result in the ability to say that ACDs have a certain percent effectiveness. Instead, this initial work will present evidence as to whether or not ACDs have the potential to be effective and safe. If positive, this data may support the implementation of ACDs in a small centre or organization to formally study the intervention in a more rigorous environment. To address the second limitation, all submissions will have a medical professional review the results prior to de-identification, and participants will be contacted if the data is inconsistent or seems medically inaccurate.

Despite its limitations, self-reporting as a sampling strategy is not new in this field of research. Redding published a case series of 225 FBAOs that were compiled by the American Heart Association through a self-reporting method [21]. This paper makes the largest single contribution to the evidence summarized in Table 2, and to ILCOR's CoSTR for FBAO interventions [6,7].

The greatest strength of this study is that it will produce the first data on ACDs that will be collected and analyzed independently of industry. Following a protocol published a priori with clearly defined outcomes and a publicly available reporting system, we aim to provide the public with information in as unbiased fashion as possible at present. In a field devoid of evidence, the first step to improving knowledge is to create a base on which to build future studies and further evaluations.

## Conclusions

This study describes a research protocol outlining the first multi-year, multi-method, global evaluation of novel suction-based airway clearance devices with the aim of improving the available data in this field and ultimately helping inform evidence-based guidelines in the future.
